# Dehydration-induced *Ae-Aper50* regulates midgut infection in *Aedes aegypti* mosquitoes

**DOI:** 10.1128/mbio.01207-24

**Published:** 2025-01-23

**Authors:** Anastasia Accoti, Margaret Becker, Angel Elma I. Abu, Julia Vulcan, Ruimei Jun, Steven G. Widen, Massamba Sylla, Vsevolod L. Popov, Laura B. Dickson

**Affiliations:** 1 Department of Microbiology and Immunology, University of Texas Medical Branch, Galveston, Texas, USA; 2 Department of Biochemistry and Molecular Biology, University of Texas Medical Branch, Galveston, Texas, USA; 3 Laboratory Vectors and Parasites, Department of Livestock Sciences and Techniques, Sine Saloum University El Hadji Ibrahima NIASS, Kaffrine Campus, Kaffrine, Senegal; 4 Department of Pathology, University of Texas Medical Branch, Galveston, Texas, USA; 5 Center for Vector-Borne and Zoonotic Diseases, University of Texas Medical Branch, Galveston, Texas, USA; 6 The West African Center for Emerging Infectious Diseases, Centers for Research in Emerging Infectious Diseases, Galveston, Texas, USA; 7 Institute for Human Infections and Immunity, University of Texas Medical Branch, Galveston, Texas, USA; Virginia Tech, Blacksburg, Virginia, USA

**Keywords:** climate change, Arbovirus, mosquito

## Abstract

Climate change is predicted to increase the spread of mosquito-borne viruses, but genetic mechanisms underlying the influence of environmental variation on the ability of insect vectors to transmit human pathogens is unknown. In response to a changing climate, mosquitoes will experience longer periods of drought. An important physiological response to dry environments is the protection against dehydration, here defined as desiccation tolerance. While temperature is known to impact interactions between mosquito and virus, the role of dehydration remains unknown. We identified two genetically diverse lines of the mosquito *Aedes aegypti*, a major arbovirus vector, with marked differences in desiccation tolerance. To determine the genetic response to dehydration between these contrasting lines, we compared gene expression profiles between desiccant- and non-desiccant-treated individuals in both the desiccation-tolerant and -susceptible lines by RNAseq. Gene expression analysis demonstrated that several genes are differentially expressed in response to desiccation stress between desiccation-tolerant and -susceptible lines. The most highly expressed transcript under desiccation stress in the desiccation-susceptible line encodes a peritrophin protein, *Ae-Aper50*. Peritrophins play a crucial role in peritrophic matrix formation in the mosquito midgut after a bloodmeal. Gene silencing of *Ae-Aper50* by RNAi demonstrated that expression of *Ae-Aper50* is required for survival of the desiccation-susceptible line under desiccation stress, but not for the desiccation-tolerant line. Moreover, the knockdown of *Ae-Aper50* resulted in higher Zika virus (ZIKV) infection rates in the desiccation-tolerant line and increased ZIKV viral replication in the desiccation susceptible line, and higher chikungunya virus (CHIKV) infection rates in the desiccation-tolerant line. Altogether, these results provide a link between protection against desiccation and midgut infection, which has important implications in predicting how climate change will impact mosquito-borne viruses.

**IMPORTANCE:**

Climate change will have profound impacts on the burden of viruses transmitted by mosquitoes. While we know how changes in temperature impact mosquito physiology and dynamics of viral replication within the mosquito, there is a complete lack of knowledge in how low humidity, or drought tolerance, will impact interactions between mosquitoes and arboviruses. Understanding how drought tolerance will alter mosquito infection with arboviruses is critical in predicting and preventing the impact that climate change will have on mosquito-borne viruses. This work demonstrates a functional link between dehydration tolerance and midgut infection. This knowledge significantly enhances our understanding of how the predicted increase in droughts could impact the dynamics of mosquito-borne viruses.

## INTRODUCTION

Climate models predict that, as global temperatures rise, the risk for arboviral disease will increase, especially in Africa (1, 2). The ongoing global expansion of Zika (3, 4) and year-round transmission potential by *Ae. aegypti* is likely to expand, particularly in South Asia and sub-Saharan Africa (1, 5). Arthropod-borne viruses (arboviruses), such as Zika, dengue, and chikungunya viruses, transmitted by the mosquito, *Aedes aegypti*, pose a major threat to public health (6). *Aedes aegypti* has an almost global distribution putting the entirety of the tropics at risk for these viruses. While most predictions about the impact of climate change on vector-borne disease transmission are focused on temperature (7–10), little is known about how other climate variables, such as humidity, will impact vector-borne disease dynamics (11, 12). Importantly, humidity is predicted to be an important climate variable involved in mosquito–pathogen interactions (13).

Like most terrestrial organisms, mosquitoes possess physiological mechanisms to prevent water loss and deal with dehydration mostly through the use of their exoskeleton (14), which contains waxes, lipids, and polysaccharides that prevent water loss across the cuticle. The cuticle composition of insects is dynamic and varies by age, metabolic status, and developmental stage (15, 16), highlighting its adaptation capacity of insects to changing environments (14, 17–19). In *Anopheles coluzzii* and *Drosophila* spp., cuticular hydrocarbons (CHCs) are the main component of the cuticle that prevents water loss (20, 21), but this has not been demonstrated for *Ae. aegypti*. Interestingly, expression of ion transporter and aquaporin-2 gene in *Ae. aegypti* has been implicated in response to dehydration (22).

Our understanding of how low humidity impacts the dynamics of vector-borne diseases remains very limited, but it is predicted to be an important variable (13). While there is limited empirical data demonstrating the impact of low humidity on vector competence (12, 23), dehydration has been shown to impact other variables contributing to overall vectorial capacity. Water loss has been directly associated with changes in mosquito behavior, leading to an increase in blood feeding frequency and therefore potential increases in transmission of West Nile virus (WNV) (24). Additionally, water loss has been connected to decreased survival and oviposition (25). Diminished nutritional resources and fecundity have been associated with alterations in both geographic and microgeographic ranges (26, 27).

In this study, we identified two genetically divergent lines of *Ae. aegypti* from Senegal (23) with marked differences in desiccation tolerance and Zika virus susceptibility. We used these lines to probe the genetic response to dehydration and identified a gene, *Ae-Aper50*, encoding a peritrophin protein that was upregulated in the desiccation-susceptible line in response to desiccation stress. Functional validation of *Ae-Aper50* confirmed its importance in desiccation tolerance in a mosquito genotype-dependent manner. We demonstrated that reduced expression of *Ae-Aper50* increases midgut infection rates in the Thiés (THI) across arbovirus families and Zika virus (ZIKV) infection intensity in the PK10 (PKT) line. Furthermore, we showed that the peritrophic matrix is thicker in the desiccation-susceptible/ZIKV-refractory line. Together, these results provide a link between the protection against desiccation and midgut infection, which has important implications in predicting how climate change will impact mosquito-borne viruses.

## RESULTS

### Desiccation tolerance is *Ae. aegypti* line dependent

**Fig 1:**
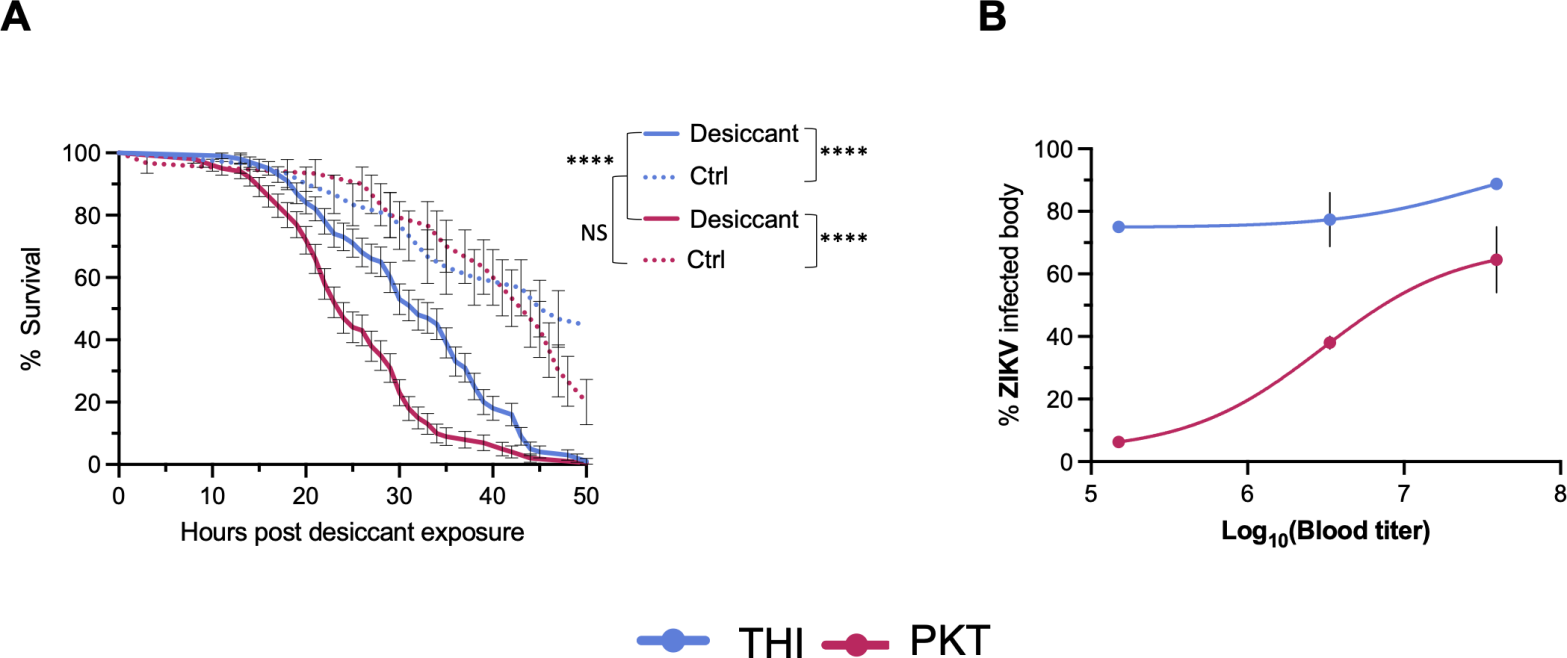
The PKT line is desiccation susceptible and less permissive to Zika virus. (**A**) Mosquitoes in single tubes were exposed to acute desiccation stress (RH 1%, 28°C) or ambient conditions (RH 50%, 28°C). Survival was video recorded for 48 h, and time of death was noted. Survival of each line and treatment was compared by a log-rank (Mantel–Cox) test. A total of 100-desiccant-treated individuals (Desiccant) and 30 untreated from two independent experiments (Ctrl) were used (THI-Desiccant vs PKT-Desiccant: *P*-value: <0.0001, THI-Ctrl vs PKT-Ctrl: *P*-value = 0.52. (**B**) Mosquitoes were infected with three different doses of ZIKV (dose I: 7.45 × 10^7^, dose II: 3.5 × 10^6^, dose III: 1.5 × 10^6^), and bodies were collected at 5 days post infectious bloodmeal. Infection rate was determined by reverse transcription-polymerase chain reaction (RT-PCR) with ZIKV-specific primers. Doses I and II represent two independent experiments with 10–38 individuals per replicate. Dose III represents a single replicate with 18–32 mosquitoes per treatment. The 50% oral infectious dose (OID_50_) for THI was 2.246 log_10_ focus-forming units (FFU)/mL of ZIKV, and the OID_50_ for PKT was 7.189 log_10_ FFU/mL of ZIKV. Overall, differences in ZIKV infection can be explained by ZIKV dose (two-way ANOVA, DF = 2, *F* = 9.61, *P*-value = 0.0297) and mosquito line (analysis of variance [ANOVA], DF = 1, *F* = 45.6, *P*-value = 0.0025).

Survival under acute desiccation was measured in two genetically divergent lines of *Ae. aegypti* originating from Senegal. Establishment and genomic characterization of the lines were done previously (28). We anticipated differences in survival under desiccation stress between the lines due to the ecology of origin for both lines. The THI line originates from the northern region of Senegal, which exhibits reduced rainfall compared to the PKT line, which originates from the southern forested region of Senegal. To determine if there are differences in desiccation tolerance between the two lines, we exposed individual females to harsh desiccation (1% relative humidity [RH]) and monitored survival for 48 h. Survival was monitored in individual females not subjected to the harsh desiccation as a control (50% RH). No difference in survival was detected between the THI and PKT lines under the control condition (Mantel–Cox, *P*-value = 0.52). Overall, the mosquitoes that were exposed to harsh desiccation survived less than the control mosquitoes (Mantel–Cox, *P*-value < 0.0001). The PKT mosquitoes died at a faster rate compared to the THI line (Mantel–Cox, *P*-value < 0.0001), with 50% of PKT mosquitoes dying within 24 h, while 50% of the population survived for 35 h in the THI line (Fig. 1A).

Previous studies have demonstrated that lines of *Ae. aegypti* from the north of Senegal are more permissive to ZIKV than lines from the south (29). To confirm this phenotype in our lines, we measured ZIKV infection rates in the THI and PKT lines following oral exposure to different doses of ZIKV. The proportion of infected individuals was determined 5 days post-infection by detecting viral RNA by RT-PCR. In accordance with previous studies, the human-adapted line of *Ae. aegypti* (THI) showed increased susceptibility to ZIKV compared to the sylvatic line (PKT) (Fig. 1B). The 50% oral infectious dose (OID_50_) of the THI line was 2.25 log_10_ focus-forming units (FFU)/mL of ZIKV compared to 7.19 log_10_ FFU/mL in the PKT line. Overall, differences in ZIKV infection can be explained by ZIKV dose (χ, *P*-value < 0.0001) and mosquito line (χ, *P*-value = 0.0128).

### Gene expression profiles under desiccation stress are *Ae. aegypti* line dependent

**Fig 2:**
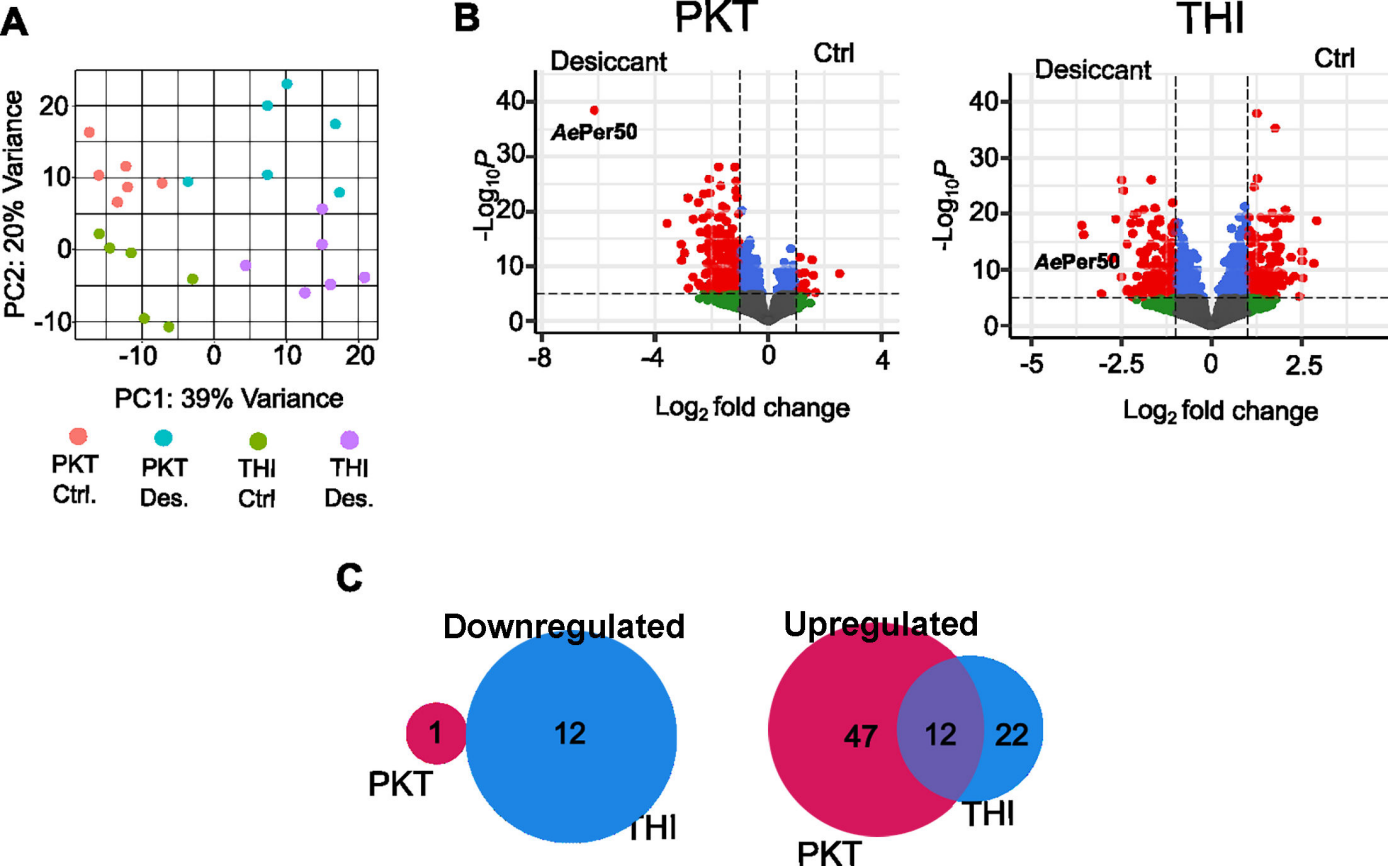
PKT and THI lines show a different genetic response to desiccation stress. Differential expression of genes was determined in response to desiccation stress by RNAseq in the PKT and THI lines. Six libraries were sequenced per treatment containing pools of five individuals. (**A**) Principal component analysis of the six sequenced libraries from each treatment. (**B**) Volcano plot of differentially expressed genes. Gray, not significant; green, log fold change greater than 1; blue, *P*-value > 0.05 red, log fold change greater than 1 and *P*-value < 0.05. (**C**) Venn diagram showing the overlap of genes either upregulated or downregulated in the THI and PKT lines during desiccation stress with a greater than twofold difference.

To determine how desiccation stress impacts gene expression in the desiccation-tolerant and -susceptible lines, we assessed the global transcriptomes of whole female bodies 24 h post 1% desiccation stress. Principal component analysis of normalized read counts of 19,804 genes showed that the sequencing libraries clustered by both mosquito line and treatment (Fig. 2A) indicating different genetic responses to desiccation between the two lines. Dehydration stress resulted in both upregulation and downregulation of differentially expressed genes. In the PKT line, a majority of the genes were upregulated in response to desiccation stress, while a similar number of genes were upregulated and downregulated in the THI line (Fig. 2B). Although the specific genes involved in dehydration tolerance have not been elucidated in *Ae. aegypti*, our data set includes genes related to chitin metabolism, lipid metabolism, and perhaps CHC biosynthesis. CHC biosynthesis occurs in oenocytes and utilizes Co-A enzymes and cytochrome p450s (30). For example, genes involved in chitin metabolism, lipid transport or lipid metabolism, and cytochrome p450 genes were identified (Tables S1 and S2). Strikingly, a single transcript encoding a peritrophin gene (AAEL002467), previously called *Ae-Aper50* (31), was highly upregulated in the PKT line. *Ae-Aper50* was also upregulated in the THI line, but not to the same magnitude.

To better understand the differential transcriptomic response between lines, we performed pair-wise comparative analysis to identify genes differentially expressed between PKT and THI lines. Of the downregulated genes with a log_2_ fold change greater than two, no genes were shared between the PKT and THI lines. Twelve genes were upregulated in both the THI and PKT lines, while each line contained upregulated genes specific to that line (Fig. 2C; Table S1). Gene ontology analysis demonstrated that genes belonging to different biological processes were upregulated in each line (Fig. S1; Table S2). In both lines, processes related to lipid transport, metabolism, and metabolic processes were identified.

### *Ae-Aper50* expression protects against desiccation in the PKT line

**Fig 3:**
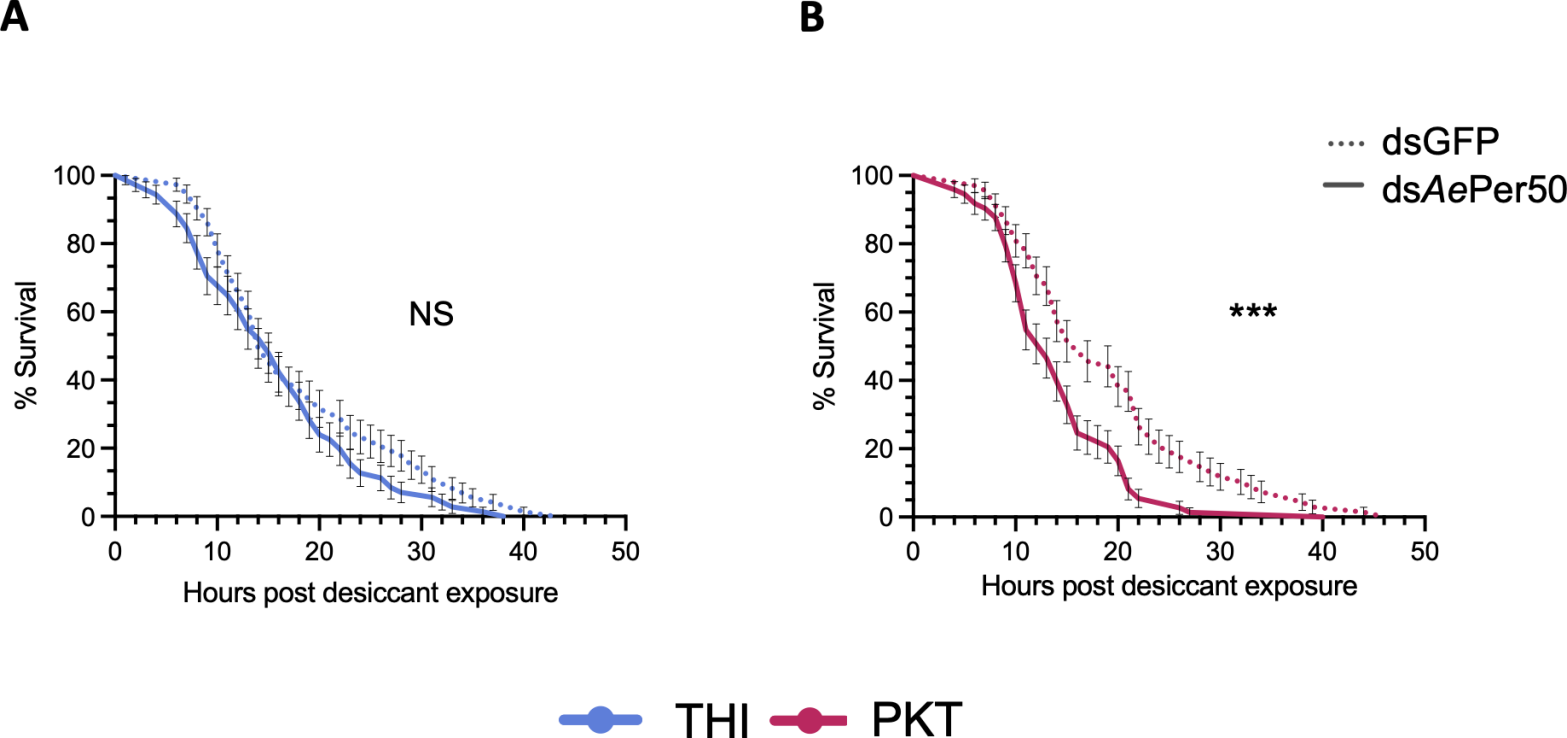
Only PKT relies on *Ae-Aper50* for protection against desiccation stress. Survival was measured in individual adult females exposed to acute desiccation stress (RH 1%) 72 h post injection with dsRNA targeting *Ae-Aper50* or GFP as a control for 48 h. Survival curves were compared by a log rank (Mantel–Cox) test on a total of 90 ds*Ae-Aper50* and 50 dsGFP-injected mosquitoes. The data represent the mean of three independent experiments; bars represent SEM (THI ds*Ae-Aper50* vs THI dsGFP: *P*-value = 0.09; PKT ds*Ae-Aper50* vs PKT dsGFP: *P*-value = 0.0002).

To confirm that *Ae-Aper50* expression plays a role in survival under desiccation stress, expression of *Ae-Aper50* was knocked down through RNAi-mediated gene silencing in both lines and survival was measured under desiccation stress. Adult female mosquitoes were injected with dsRNA targeting *Ae-Aper50* and dsRNA targeting GFP as control. Survival was monitored for 48 h following exposure to 1% RH for 72 h post injection. Knockdown of *Ae-Aper50* did not affect survival under desiccation stress in the THI line (Mantel–Cox, *P*-value = 0.09) (Fig. 3A). In the PKT line, the knockdown of *Ae-Aper50* resulted in a reduced lifespan compared to the dsGFP-injected control (Mantel–Cox, *P*-value = 0.0002) (Fig. 3B). Knockdown of *Ae-Aper50* was confirmed 72 h post-injection by qPCR (Fig. S2).

### *Ae-Aper50* is expressed in the midgut following a bloodmeal

Expression of the *Ae-Aper50* gene has previously been described as being restricted to the midgut following a bloodmeal (31). To confirm that expression of *Ae-Aper50* is also induced following a bloodmeal in our lines, expression of *Ae-Aper50* was measured by qPCR following a bloodmeal. In accordance with previously published work, we saw induction of *Ae-Aper50* expression within 4 h following a bloodmeal, then diminishing by 12 h following a bloodmeal in both the THI and PKT lines (Fig. S3).

### PKT has a wider peritrophic matrix than THI

**Fig 4:**
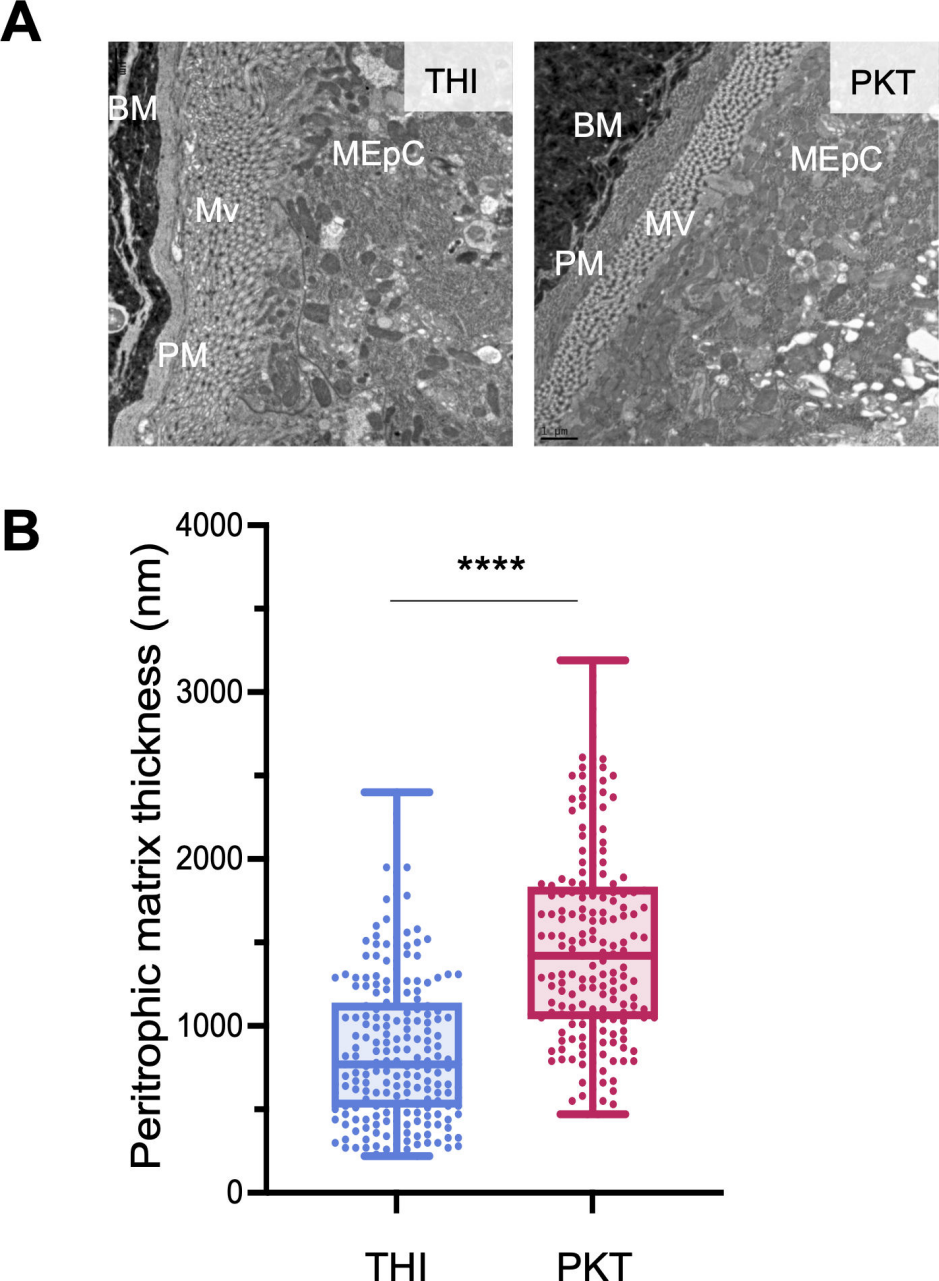
PKT has a thicker peritrophic matrix compared to THI. Electron microscopy was used to capture images of the peritrophic matrix in either the PKT or THI line 22 ± 0.5 h post bloodmeal. (**A**) Representative electron microscopy image of a longitudinal midgut section (80 nm) through the center of the midgut from each line. BM, bloodmeal; PM, peritrophic matrix; Mv, microvillae; MEpC, midgut epithelial cell. (B) The thickness was measured at a minimum of 10 spots around the entire peritrophic matrix perimeter avoiding the breakage; each dot represents a single measurement. The thickness of the peritrophic matrix was compared between THI and PKT with an unpaired non-parametric *t*-test Mann–Whitney (*P*-value = <0.0001).

Peritrophins, and specifically *Ae-Aper50*, have been previously characterized for their presence in the peritrophic matrix (32), a chitinous sac that envelops the blood bolus in the posterior midgut following a bloodmeal. Given that we saw differences in infection rates between the THI and PKT lines, ultrastructural images of blood-fed midguts were taken using transmission electron microscopy to visualize any structural differences in the peritrophic matrix between lines. The peritrophic matrix surrounding the blood bolus was measured in 10 locations around the midguts being sure to avoid areas of breakage (Fig. 4A). The more ZIKV-refractory line (PKT) had a thicker peritrophic matrix (2,136.0 ± 964.6 nm) compared to the more susceptible line (THI) (1,062.0 ± 547.2 nm) (Mann–Whitney test, *P*-value of <0.0001) (Fig. 4B). To ensure that the difference in thickness was not biased by the overall size of the midgut from each *Ae. aegypti* line, the length and area of each midgut was analyzed and recorded in the midgut sections. Overall, the THI midgut were larger than the PKT midguts, but no statistical differences in the length or area were detected (Fig. S4).

### *Ae-Aper50* expression controls midgut infection with ZIKV and CHIKV

**Fig 5:**
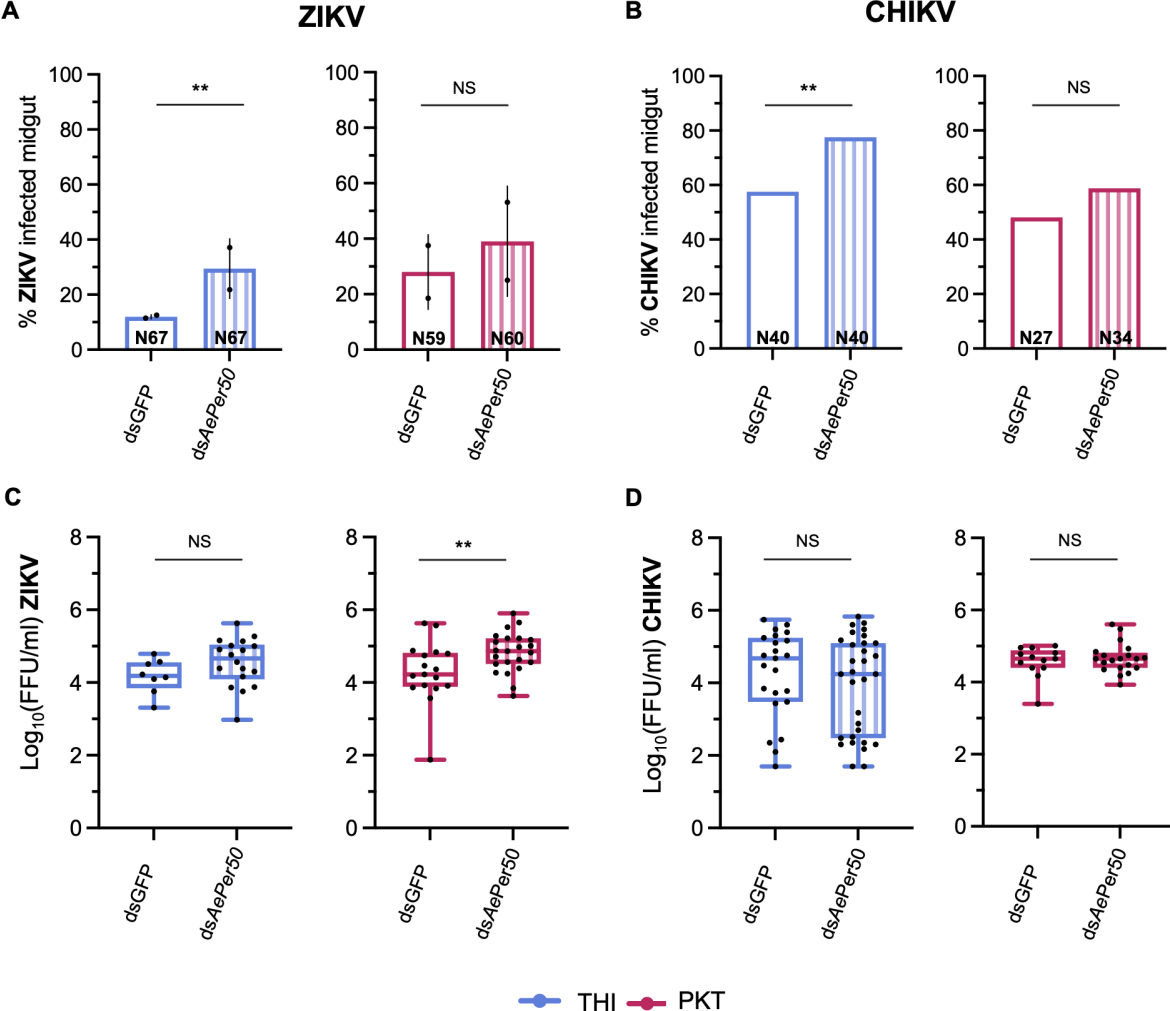
Expression of *Ae-Aper50* regulates arbovirus infection. The proportion (**A, B**) of infected *Ae. aegypti* midguts and the amount of infectious virus (**C, D**) in *Ae. aegypti* midguts from the THI and PKT lines exposed to ZIKV (**A, C**) or CHIKV (**B, D**) following infection with dsRNA targeting either *Ae-Aper50* or GFP as a control. Midguts were dissected 5 days post infectious bloodmeal, and proportion of infected midguts and viral titers in the midgut were determined by focus forming unit assay. (**A, B**) Bar graph showing the proportion of infected midguts with either ZIKV or CHIKV following knockdown of *Ae-Aper50*. The proportion of infected midguts was compared by χ analysis of a binomial logistic regression as a function of infection; error bars represent the SEM of the proportions (for ZIKV, THI: ds*Ae-Aper50* vs dsGFP *P*-value = 0.007; PK10: ds*Ae-Aper50* vs dsGFP *P*-value > 0.05) (for CHIKV, THI: ds*Ae-Aper50* vs dsGFP *P*-value = 0.048 ; PK10: ds*Ae-Aper50* vs dsGFP *P*-value > 0.05). (**C, D**) Boxplot showing the titers of infectious ZIKV (**C**) and CHIKV (**D**) particles in the midgut 5 days post infection. Each point represents an individual midgut, and the mean is represented by a horizontal line. The error bars represent the 95% CI. Data were analyzed by two-way ANOVA as a function of infection (for ZIKV, THI: ds*Ae-Aper50* vs dsGFP *P*-value > 0.05; PK10: ds*Ae-Aper50* vs dsGFP *P*-value = 0.019) (for CHIKV, THI: ds*Ae-Aper50* vs dsGFP *P*-value > 0.05; PK10: ds*Ae-Aper50* vs dsGFP *P*-value > 0.05). Data represents two independent replicates for the ZIKV infection and one replicate for the CHIKV infection. The total number of individuals tested is displayed on each bar. THI and PKT experiments were done with different stocks of ZIKV (see Materials and Methods for details.

To determine if *Ae-Aper50* plays a role in midgut permissiveness to arboviruses, the THI and PKT lines were injected with dsRNA targeting *Ae-Aper50* or GFP as a control. Adult females were offered an artificial bloodmeal containing either ZIKV or chikungunya virus (CHIKV) 72 h post-injection. Given that *Ae-Aper50* is highly expressed following a bloodmeal (31), we measured the expression 24 h following a bloodmeal and confirmed knockdown of *Ae-Aper50* (Fig. S2). Given that we observe differences in infection rates when fed the same titer between the two lines, PKT and THI were fed different doses of ZIKV to target similar infection rates. Midgut infection rates and viral titers (FFU/mL) were assayed 5 days post-virus exposure in dissected midguts. In the THI line, 12% of dsGFP-injected mosquitoes became infected with ZIKV, while 30% of ds*Ae-Aper50*-injected mosquitoes became infected (χ test, *P*-value = 0.007) (Fig. 5A). Although the ds*Ae-Aper50*-injected THI mosquitoes had more infectious ZIKV particles in the midgut compared to the dsGFP-injected mosquitoes, this difference was not statistically significant (Fig. 5B). In the PKT line, knockdown of *Ae-Aper50* resulted in a higher rate (37.5%) of ZIKV infection compared to the control (29.8%), but this difference was not statistically significant (Fig. 5A). Knockdown of *Ae-Aper50* resulted in more infectious ZIKV particles in the midgut compared to the controls in the PKT line (ANOVA, *P*-value = 0.019) (Fig. 5B). See Materials and Methods for information on bloodmeal titers used between the THI and PKT lines.

To determine if the importance of *Ae-Aper50* is generalizable across arbovirus families, we challenged mosquitoes with CHIKV following knockdown of *Ae-Aper50*. We found a similar trend, with a higher proportion of mosquitoes becoming infected following knockdown of *Ae-Aper50* compared to the control in both lines (77.5% vs 57.5% in THI, and 58.8% vs 48.1% in PKT), although a significant difference in infection rates was only observed in the THI line (THI *P*-value = 0.048) (Fig. 5C). No differences in the number of CHIKV infectious particles were detected in the midgut following *Ae-Aper50* knockdown in either line (Fig. 5D).

## DISCUSSION

In this study, we used two genetically divergent *Ae. aegypti* lines, which display differences in survival under desiccation stress, to probe the genetic response of mosquitoes to dehydration. We first identified two lines of *Ae. aegypti* that exhibit differences in survival under desiccation stress. In accordance with previously published results, these lines also display differences in ZIKV infection rates. Transcriptomics was used to identify differences in the genetic response to dehydration between the lines. The most highly expressed gene under desiccation stress in the desiccation-susceptible line encodes a peritrophin protein, *Ae-Aper50*. Knockdown of *Ae-Aper50* expression confirmed its role in protection against desiccation in the desiccation-susceptible line, but not the desiccation-tolerant line. Because peritrophin proteins are known to be involved in forming the peritrophic matrix, we measured the influence of this gene on midgut infection. Expression of *Ae-Aper50* was important for reducing ZIKV and CHIKV infection rates in THI line and ZIKV infection intensity in the PKT line highlighting the importance of this gene across virus families. Together, these data demonstrate that differences in desiccation tolerance are functionally linked to the process surrounding midgut infection, which has important implications for predicting how climate change will impact mosquito-borne virus transmission.

An important finding of this study is the difference in the genetic response to desiccation between the two lines. Mosquitoes avoid desiccation by preventing water loss across their cuticle. The cuticle is composed of lipids, waxes such as CHCs, and polysaccharides such as chitin. It is expected that the differential production of CHCs is the main mechanism for regulation of desiccation tolerance in mosquitoes given their importance in other insects (20, 21), but it is possible that genes in other pathways also contribute to desiccation tolerance. While the specific genes involved in desiccation tolerance in *Ae. aegypti* have not been described, our transcriptomics data set identified multiple genes related to lipid metabolism and transport, synthesis of CHCs (14 cytochrome p450 genes in the THI line and seven in the PKT line), and chitin-binding genes (*Ae-Aper50* AAEL002467 and AAEL006159). Our data suggest that the two lines possess different mechanisms to respond to dehydration. This could have arisen from differences in the ecology and climate where these lines originated (28). The THI line originates from a drier environment than the PKT and could have evolved mechanisms to withstand desiccation that the PKT line has not acquired, or has lost. Perhaps adapting away from using *Ae-Aper50* for desiccation protection is linked to a thinning of the PM. Additional work needs to be done to elucidate the mechanisms of desiccation tolerance between these two lines.

The involvement of *Ae-Aper50* during desiccation stress could be related to its intrinsic ability to stabilize chitin (31), which is a component of the insect peritrophic matrix as well as a major component of the insect cuticle (33). Previous work has shown that *Ae-Aper50* is only expressed in the midgut following a blood meal and to a lesser extent after sugar feeding (31). Additionally, Shao et al. did not detect *Ae-Aper50* mRNA in blood- or sugar-fed carcasses suggesting that expression is restricted to the midgut, but expression in the tissue outside the midgut has not been tested under desiccation stress. Here, RNAseq was done on whole bodies, so we cannot conclude whether *Ae-Aper50* expression is occurring in the carcass or the midgut during desiccation stress. Although previous studies have shown that *Ae-Aper5*0 is restricted to the midgut following a bloodmeal, we cannot conclude where it is being expressed in this study. Given the absence of a bloodmeal, there should be no formation of the peritrophic matrix under desiccation stress, and we anticipate that the mechanism of *Ae-Aper50* under desiccation stress is independent of the peritrophic matrix. Further experimental work needs to be done to localize the expression of *Ae-Aper50* under desiccation stress and work out the mechanism.

*Ae-Aper50* belongs to the class of peritrophins that have chitin-binding motifs containing amino acid sequences consisting of six spaced cysteines referred to as the “peritrophin A domain” (31). This domain is found in numerous proteins extracted from insect peritrophic matrices (34). In contrast, different chitin-binding motifs in peritrophin proteins are found in the insect cuticle (35). Given that *Ae-A*per50 belongs to the class of peritrophins found in the peritrophic matrix, it is interesting that we find it highly expressed in the whole body independent of a bloodmeal and the peritrophic matrix formation. Again, further work needs to be done to define the localization and mechanism of *Ae-Aper50* under desiccation stress.

One of the main functions of the peritrophic matrix is protecting the mosquito from pathogen invasion (36–38). Secretion of the peritrophic matrix in *Ae. aegypti* begins approximately 4 h after the bloodmeal, and it becomes separated from the midgut epithelial cell microvilli at 8 to 12 h after blood feeding (39). Our data indicate that reduced levels of *Ae-Aper50* expression increase both midgut infection and replication of ZIKV as well as midgut infection of CHIKV. Reducing the expression of Ae-Aper50 could increase midgut infection through disruption of the peritrophic matrix. The importance of this gene in controlling infection across viral families suggests that *Ae-Aper50* may play a structural role in the peritrophic matrix that is important for controlling access to the midgut epithelium, as opposed to a mechanism that involves viral tropism.

Here, we identified differential thickness of the peritrophic matrix between the THI and PKT lines. These differences in the peritrophic matrix thickness were observed following a bloodmeal and in the absence of desiccation stress. We are not claiming that peritrophic matrix thickness contributes to desiccation tolerance given that there is no bloodmeal present, and the peritrophic matrix should not be formed. Specifically, we found that the desiccation-susceptible/ZIKV-refractory line has a thicker peritrophic matrix than the desiccation-tolerant/ZIKV-permissive line. It is tempting to speculate that the differences in ZIKV infection rates between the THI and PKT lines are due to the width of the peritrophic matrix. This is supported by the fact that we can see a significant increase in ZIKV infection rates in the THI upon silencing *Ae-Aper50*, but not in the PKT line. Perhaps this is due to the fact that the peritrophic matrix of THI is thinner to start, and we were able to reduce the thickness to a meaningful level with our gene silencing assay, which is not the case with the thicker PKT line. Given that we see a trend in increased infection rates after silencing *Ae-Aper50* in the PKT line, it is likely that a higher degree of gene silencing would allow for complete thinning of the peritrophic matrix and a significant increase in midgut infection rates.

We also observed the role of *Ae-Aper50* in rates of ZIKV replication in the midgut only in the PKT line. A significant difference in the amount of replicating ZIKV viral particles between dsGFP and ds*Ae-Aper50*-treated mosquitoes was only observed in the PKT line. This suggests that a function of *Ae-Aper50* outside of the thickness of the PM regulates rates of viral replication, perhaps due to the number of virus particles, which access the midgut epithelium. No differences in the amount of replicating CHIKV were found between dsGFP and ds*Ae-Aper50*-treated mosquitoes in both lines suggesting that PM features that regulate viral access to the midgut and subsequent replication differ between CHIKV and ZIKV.

*Ae-Aper50* could be impacting midgut infection through other mechanisms besides establishing the thickness of the peritrophic matrix. Several factors have been shown to influence the ability of a virus to escape from the blood bolus to the epithelial cells, referred to as the midgut infection barrier (39). Although we observed a thicker peritrophic matrix in the PKT line, there are other possible explanations for why this line is more refractory than the THI line. Differences in timing of *Ae-Aper50* expression and peritrophic matrix formation, the composition of the peritrophic matrix (how much *Ae-Aper50* protein is in the peritrophic matrix), or the permeability/stability of the peritrophic matrix could also impact the ability of the virus to reach the midgut epithelium. The thicker peritrophic matrix in the PKT line could be a result of differential timing of formation, resulting in an earlier and longer PM secretion that results in a wider peritrophic matrix. We did not investigate the PM composition in the *Ae. aegypti* lines we used in this study, and this is worthy of further investigation. Perhaps the PKT line has a higher density of the *Ae-Aper50* protein in the peritrophic matrix resulting in a stronger barrier for arboviral infection. Additional studies of the kinetics of PM formation in these two mosquito lines are needed to better understand likely mechanisms of midgut infection and replication.

Overall, this study demonstrates a link between dehydration tolerance and midgut infection which has important implications for predicting how climate change will impact mosquito-borne virus transmission.

## MATERIALS AND METHODS

### *Aedes aegypti* mosquitoes

Colonies of *Ae. aegypti* used in this study originated from a natural population of THI and PKT originally sampled, respectively, in the area of Thiès and the PK10 forest in Senegal (28). Colonies were established by collecting eggs from each colony using ovitraps as described by Rose et al. (28). The mosquitoes of the study belong to generation 9 or 10 depending on the experiment. Mosquitoes were reared under standard insectary conditions consisting of 28°C and 70% ± 10% relative humidity with a 12 h:12 h light–dark cycle. Eggs were hatched in deionized water, and larvae were fed fish food. Adults were held in BugDorm cages with constant access to 10% sucrose until being used for specific experimental procedures.

### Desiccation assay

To determine the acute desiccation tolerance, the method previously described by (40, 40) was adapted as follows. Individual 4- to 5-day-old female mosquitoes from both the THI and PKT colonies were cold anesthetized and placed into plastic *Drosophila* vials (Flystuff Narrow Drosophila Vials, Polystyrene) and plugged with a foam stopper (Flystuff 59-200 Droso-Plugs) approximately 2 cm below the rim of the vial. After all mosquitoes were added to the vials, a desiccation agent (Drierite Mesh size 8) was added on top of the foam stopper, and parafilm was used to seal the vial. The amount of desiccant agent added was normalized using a scoop with a volume of approximately 6 g of desiccant agent. Controls had no desiccant agent added and were sealed with parafilm as the treated mosquitoes. Neither the controls nor the desiccant-treated mosquitoes had access to sugar or water. The desiccant agent reduced the relative humidity within the vials to <1%, and the control vials resulted in an RH of 50% (Onset HOBO External Temp/RH Data Logger). The experiment started upon sealing the vials and was terminated after 48 h. Vials were placed on shelves with approximately 50 vials per shelf inside an incubator (Thermo Fisher-Precision) set to 28°C with a 12-h light–dark cycle. To monitor the status of the mosquitoes, home security cameras (Wyze Cam v3) were used. Each camera captured the activity of approximately 50 mosquitos with an HD resolution of 1,920 × 1,080 pixels at 20 Hz during the light cycle and 15 Hz during the dark cycle. The cameras use infrared light to record during the dark cycle. The time of death for each individual mosquito was estimated by watching a footage of the beginning of each hour over the study period. Mosquitos were considered dead when they were knocked down or otherwise immobile. For every line, a total of 50 individuals for the desiccant and 15 for the control were used in two separate experiments. The statistical significance of survival curves was set to the conventional α < 0.05 level, calculated with a log-rank Mantel–Cox analysis and using GraphPad Prism software, version 10.

### Vector competence

THI and PKT females, 4 to 5 days old, were starved for 24 h before the infectious blood meal. Vector competence assays were performed in an arthropod containment level 2 facility (ACL-2). Briefly, mosquitoes were experimentally exposed to different titers of wild-type Zika virus Cambodia isolate (FSS 13052) received from the World Reference Center for Emerging Viruses and Arboviruses at UTMB. The virus stock was diluted in cell culture media (Dulbecco’s modified Eagle medium [DMEM] with the addition of 1.5% heat-inactivated fetal bovine serum [FBS], 1% penicillin/streptomycin) and 30 µL of 7.5% sodium bicarbonate to reach a dose of 7.5 × 10^7^ (dose I), 3.5 × 10^6^ (dose II), and 1.5 × 10^5^ (dose III) FFU/mL. One volume of virus suspension was mixed with two volumes of defibrinated sheep blood (Colorado Serum Company) washed three times in 1× phosphate-buffered saline (PBS) and 60 µL of 100 mM adenosine 5′-triphosphate. After gentle mixing, 2 mL of the infectious blood meal was added to Hemotek membrane feeders (Hemotek Ltd.) covered with a piece of desalted porcine intestine as a membrane and maintained at 37°C. After feeding, fully engorged females were sorted into 1-pint cardboard cups and maintained under controlled conditions (28 ± 1°C; relative humidity, 75% ± 5%; 12:12 h light/dark cycle) in a climatic chamber for 5 days. After 5 days of incubation, whole bodies of ZIKV-exposed mosquitoes were harvested in 2-mL screw-lid vials and homogenized in 300 µL of crude RNA extraction buffer/squash buffer (10 mM Tris HCl, 50 mM NaCl, 1.25 mM EDTA, fresh 0.35 g/L proteinase K) in a Precellys homogenizer for three rounds of 20 s with a 30-s pause every round. After homogenization, 200 µL of each sample was transferred to a 96-well plate and incubated at 56°C for 5 min followed by 98°C for 10 min. cDNA was produced from 5 µL of each sample using M-MLV reverse transcriptase (Invitrogen) and random hexamers by the following program: 10 min at 25°C, 50 min at 37°C, and 15 min at 70°C. The cDNA (2.5 µL) was amplified by PCR carried out with DreamTaq DNA polymerase (Thermo Fisher) and specific ZIKV primers (forward: 5′-GTATGGAATGGAGATAAGGCCCA-3′, and reverse: 5′-ACCAGCACTGCCATTGATGTGC-3′) (29). Amplicons were visualized by gel electrophoresis on a 2% agarose gel. The proportion of ZIKV-infected females was analyzed by χ analysis on a binomial logistic regression as a function of treatment and colony in R. For doses I and II, two independent biological replicates were conducted, with a total of 70 individuals for THI and 43 for PKT and 53 individuals for THI and 24 for PKT, respectively. For dose III, a single replicate was carried out, with 32 individuals for THI and 18 for PKT.

### RNA sequencing

THI and PKT females, 4 to 5 days old, were exposed for 24 h to 1% of relative humidity (RH); following the treatment, whole bodies were homogenized in 800 mL of TRIzol (Invitrogen), and RNA was extracted with the phenol–chloroform method following the manufacturer’s instruction. The final resuspension of RNA was in 20 µL of nuclease-free water.

The UTMB Next Generation Sequence (NGS) core laboratory assessed RNA concentrations and quality using a Nanodrop ND-1000 spectrophotometer (Thermofisher, Waltham) and an Agilent Bioanalyzer 2100 (Agilent Technologies, Santa Clara, CA). PolyA+ RNA was purified from ~100 ng of total RNA, and sequencing libraries were prepared with the NEBNext Ultra II RNA library kit (New England Biolabs) following the manufacturer’s protocol. Libraries were pooled and sequenced on an Illumina NextSeq 550 High Output flow-cell with a paired-end 75 base protocol.

STAR alignment software, version 2.7.10a, was used to build a genome index and map the reads. The index was built from the *Ae. aegypti* LVP_AGWG genome and annotation files downloaded from VectorBase.org, release 59. Reads were mapped with the parameters recommended for the ENCODE consortium, and reads mapping to genes were quantified with the STAR–quantMode GeneCounts option. Differential gene expression was estimated with the R v4.1.3 DESeq2 software package, version 1.32.0, following the vignette provided with the package. Log fold changes were moderated with the lfcShrink function using the ashr package.

### Gene enrichment analysis

Significant genes with at least a twofold difference in expression were analyzed for functional enrichment by submitting the gene lists to VectorBase Release 65. Enriched gene ontology (GO) terms were identified using the *Ae. aegypti* LVP_AGWG background and clustered with REVIGO at a medium similarity using *Drosophila melanogaster* as the closest species in the database.

### *Ae-Aper50* gene expression time course

To monitor the line-specific *Ae-Aper50* gene expression, 4- to 5-day-old female *Aedes aegypti* THI and PKT lines were blood fed a non-infectious bloodmeal, and midguts were collected at different time points. Mosquitoes were starved 24 h before the blood feeding. Feeders were prepared, as described in a previous section, on the day of the feed. Mosquitoes were fed for 30 min, and the fully engorged were quickly sorted on ice. Midguts were dissected at 4, 8, 12, 24, and 48 h post bloodmeal, and midguts from unfed females were used as control. Midguts were placed in vials containing 0.1-mm glass beads and 200 µL of TRIzol (Invitrogen), homogenized after collection and stored at −80°C until processing. RNA was extracted using a Quick-RNA Miniprep kit (Zymo). *Ae-Aper50* reverse transcription (RT) and qPCR reactions were performed using GoTaq 1-Step RT-qPCR System (Promega), with 10 µL of 2× qPCR Master Mix, 1 µL of 10 µM primer and 3 µL of cDNA and 0.4 µL of RT enzyme, made up to a total reaction volume of 20 µL with nuclease-free water. Primers used were either *Ae-Aper50* primers (FWD-TCATCCTCACCTTCGCCTAC, REV-AAGCTCTGTCGTCGTTGTGGG) or S7 primers (FWD-GCA GAC CAC CAT TGA ACA CA, REV-CAC GTC CGG TCA GCT TCT TG) as a housekeeping gene. Positive control and no template control were added to every qPCR run to assess for contamination and cross primer-dimer formation. The *Ae-Aper50* qPCR was performed on a QuantStudio 6 Real-Time PCR System (Applied Biosystems), with cycling conditions as follows: initial denaturation at 95°C for 20 s and 45 cycles at 95°C for 10 s and 56°C for 30 s, and the melting curve 95°C for 10 min, 65°C to 95°C with an increment of 0.5°C every 0.05 s. All qPCRs were performed in duplicate, and the expression levels of target genes were normalized to the levels of ribosomal protein S7. The fold change in the gene expression was calculated according to the standard DDCT method, using the unfed midgut as the control. For every mosquito line, a total of 12 midguts per time point were processed.

### Gene silencing assay

For producing *Ae-Aper50* line-specific dsRNA, total RNA was extracted with Quick-RNA Miniprep kit (Zymo Research) from 10 whole nonblood-fed (NBF) *Ae. aegypti* females according to manufacturer’s instructions including DNase treatment (Zymo Research). cDNA was generated from 1  µg of total RNA using M-MLV reverse-transcriptase (Invitrogen) and random hexamers (Invitrogen). cDNA was used for the production of the dsRNAs targeting *Ae-Aper50* THI- and PKT-gene, and the plasmid template *pUC57* (Addgene) was used for dsGFP (used as control dsRNA). dsRNAs were produced as previously described (41) using primers with T7 RNA polymerase promoter sequence (42). *Ae-Aper50* dsRNA primers were designed using the E-RNAi web service (https://www.dkfz.de/signaling/e-rnai3/0). RNA interference assays (RNAi-based gene silencing) were conducted as previously reported (41). Briefly, 69 nL of dsRNA (3 µg/µL) re-suspended in water was injected into the thorax of cold-anesthetized 3- to 4-day-old female mosquitoes using a Nanoject III (Drummond Scientific).

To evaluate the knockdown efficiency, we measured *Ae-Aper50* expression at two time points, whole bodies at 72 h post injection and midguts at 96 h post injection/24 h post bloodmeal. We checked whole bodies to confirm knockdown in our desiccation survival assay and checked midguts 96 h post injection/24 h post bloodmeal to ensure *Ae-Aper50* was still being silenced despite induction of the gene following a bloodmeal. RNA extraction, RT, and qPCR were performed as described in the previous section, and the average of fold changes was used to calculate the percentage of knockdown. Knockdown efficiency was determined by normalizing the fold change of *Ae-Aper50* relative to S7 housekeeping genes in the ds*Ae-Aper50*-injected mosquitoes to dsGFP-injected mosquitoes.

### Arboviral infection and focus forming assay on *Ae-Aper50*-injected *Ae. aegypti*

Mosquitoes, 72 h post dsRNA injection, performed as described in the previous section, were infected with the wild-type Zika virus Cambodia isolate (FSS 13025) or the wild-type chikungunya virus isolate (FSS 37997, Senegal, mosquito, 1983, West African lineage) received from the World Reference Center for Emerging Viruses and Arboviruses at UTMB. The infectious bloodmeal was prepared as described before. Initial experiments confirmed that the dsGFP-injected THI line was more susceptible than the dsGFP PKT line (Fig. S5). Differences between dsRNA treatments were difficult to observe given the high infection rates, so the assays were repeated at lower titers. The stock of ZIKV ran out following the assays in the PKT line, and a new stock of ZIKV was used for the THI lines. Two replicates were performed in each line and fed a titer of 2.3 × 10^6^ and 3.5 × 10^6^ FFU/mL for the THI line (Stock B) and of 4 × 10^6^ and 3.6 × 10^6^ FFU/mL for the PKT line (Stock A). Differences in the infectivity of Stocks A and B likely explain the lower infection rates in the THI line compared to the PKT line for the knockdown assays. For the CHIKV infection, the blood meal titer was 7 × 10^6^ FFU/mL for both *Ae. aegypti* lines. To assess the infection rate of the midguts, a focus-forming assay in Vero cells was performed. Briefly, midguts were homogenized individually in vials containing 0.1 mm glass beads and 200 µL of Vero cell media, which consist of DMEM with 1% penicillin/streptomycin (Pen-Strep), 1% antibiotic and antimycotic (GIBCO), and 5% heat-inactivated FBS. Samples were stored at −80°C until processed. Vero cells were seeded in 24-well plates and incubated for 48 h to reach confluency. Each well was inoculated with 200 µL of midgut homogenate in 10-fold dilutions (from 10^1^ to 10^4^) and incubated at 37°C (5% CO_2_) for 1 h, rocking every 15 min. Infected cells were overlaid with OPTI-MEM media supplemented with 1.25% carboxymethyl cellulose, 5% FBS, and 1% Pen-Strep. After 3 days of incubation at 37°C, infected cells were fixed with 10% formalin for at least 1 h and were washed three times in 1× PBS. Approximately 500 µL of blocking solution (5% [wt/vol] non-fat powdered milk in 1× PBS) was added to each well, and plates were rocked for 30 min. The blocking solution was discarded, and 200 µL of primary antibody solution (obtained from the World Reference Center for Emerging Viruses and Arboviruses-WRCEVA at UTMB) diluted 1:1,000 in blocking solution was added to each well, and plates were placed on a plate rocker overnight. The primary antibody solution was discarded, and plates were washed three times with 1× PBS prior to the addition of 200 µL of secondary antibody (peroxidase-labeled goat anti-mouse IgG human serum KPL-474-1806) solution diluted 1:2,000 in blocking solution. Plates were placed on a plate rocker for 1 h. The secondary antibody solution was discarded, and plates were washed three times with 1× PBS. To develop visible foci, 100 µL of TrueBlue peroxidase substrate (KPL 5510-0050) was added to each well, and plates were placed on the plate rocker until foci could be seen approximately 10 min. Plates were washed with deionized water, and FFU was counted with the help of a light. Focus-forming units were log_10_ transformed to represent the concentration of infectious ZIKV particles detected in *Ae. aegypti* midguts. For the ZIKV experiment, two independent experiments were performed, and per each knock down treatment (dsGFP and *dsAe-Aper50*). THI had 32 ± 3 and PKT had 32 ± 4. For the chikungunya experiment, one experiment was performed, and per each treatment, THI had 40 and PKT had 34 ± 7. Data were analyzed by two-way ANOVA as a function of infection in R.

### Peritrophic matrix electron microscopy

For ultrastructural analysis of ultrathin sections, THI and PKT females 4 to 5 days old were starved for 24 h before the blood meal, which consists of 2 mL of de-fibrinated sheep blood (Colorado Veterinary Product) washed three time in 1× PBS with the addition of 60 µL of 100 mM adenosine 5′-triphosphate. Only fully engorged mosquitoes were sorted into 1-pint cardboard cups and maintained under controlled conditions (28 ± 1°C; relative humidity, 75% ± 5%; 12:12 h light/dark cycle) in a climatic chamber for 22 ± 0.5 h when they were carefully dissected in 1× PBS avoiding pulling the midgut. After collection, each midgut was placed in 1 mL of primary aldehyde fixative and stored at 4°C for no longer than 1 month (28). For ultrastructural analysis in ultrathin sections midguts were fixed in primary fixative—a mixture of 2.5% formaldehyde prepared from paraformaldehyde, 0.1% glutaraldehyde, 0.01% picric acid, and 0.03% CaCl_2_ in 0.05 M cacodylate buffer (pH 7.3). They were washed in 0.1 M cacodylate buffer, post-fixed in 1% OsO_4_ in 0.1 M cacodylate buffer (pH 7.3) for 1 h, washed with distilled water and *en bloc* stained with 2% aqueous uranyl acetate for 20 min at 60°C. Then, they were dehydrated in ascending concentrations of ethanol, processed through propylene oxide, and embedded in Poly/Bed 812 (Polysciences, Warrington, PA).

Before cutting ultrathin sections, 1 μm semi-thin sections were cut and stained with toluidine blue on glass slides. Semi-thin and ultrathin sections were cut on Leica EM UC7 ultramicrotome (Leica Microsystems, Buffalo Grove, IL). Longitudinal ultrathin sections were placed on Formvar-coated 2 × 1 mm slotted copper grids (FF2010-Cu-50, Electron Microscopy Sciences, Hatfield, PA), stained with lead citrate, and examined in a JEM-1400 (JEOL USA, Peabody, MA) transmission electron microscope at 80 kV. Digital images were acquired with a bottom-mounted CCD camera Orius-SC200-1 (Gatan, Pleasanton, CA). A minimum of 10 pictures were taken along the whole midgut perimeter in areas without peritrophic matrix detachment. Measurements were taken with Gatan software in the electron microscope.

To avoid bias related to the size of the midgut, the peritrophic matrix size measurements were taken together with whole midgut size and area from 1 µm toluidine-blue-stained semithin sections using NIS-Element Viewer software. For every mosquito line, three independent experiments were performed, one midgut per replicate. The mean width of the peritrophic matrix was compared between the THI and PKT lines with a Mann–Whitney unpaired *t*-test in Graphpad Prism (Version 10).

For every mosquito line, three independent experiments were performed, one midgut per replicate. The mean width of the peritrophic matrix was compared between the THI and PKT lines with a Mann–Whitney unpaired *t*-test in Graphpad Prism (Version 10).

## Data Availability

Data are available through accession number GSE282983.

## SUPPLEMENTAL MATERIAL

Supplemental Figures mbio.01207-24-s0001.docx Figures S1 to S5 and captions to supplemental tables.

Supplemental Tables mbio.01207-24-s0002.pdf Tables S1 and S2.

ASM does not own the copyrights to Supplemental Material that may be linked to, or accessed through, an article. The authors have granted ASM a non-exclusive, world-wide license to publish the Supplemental Material files. Please contact the corresponding author directly for reuse.
