# Supplementary material for: Dehydration-induced Ae-Aper50 regulates midgut infection in Aedes aegypti mosquitoes
Source: mBio. 2025 Jan 23;16(3):e01207-24. doi: 10.1128/mbio.01207-24 (PMC11898677; doi:10.1128/mbio.01207-24)
Supplement: Supplemental Figures — Figures S1 to S5 and captions to supplemental tables. [file mbio.01207-24-s0001.docx]

**Supplemental Information**


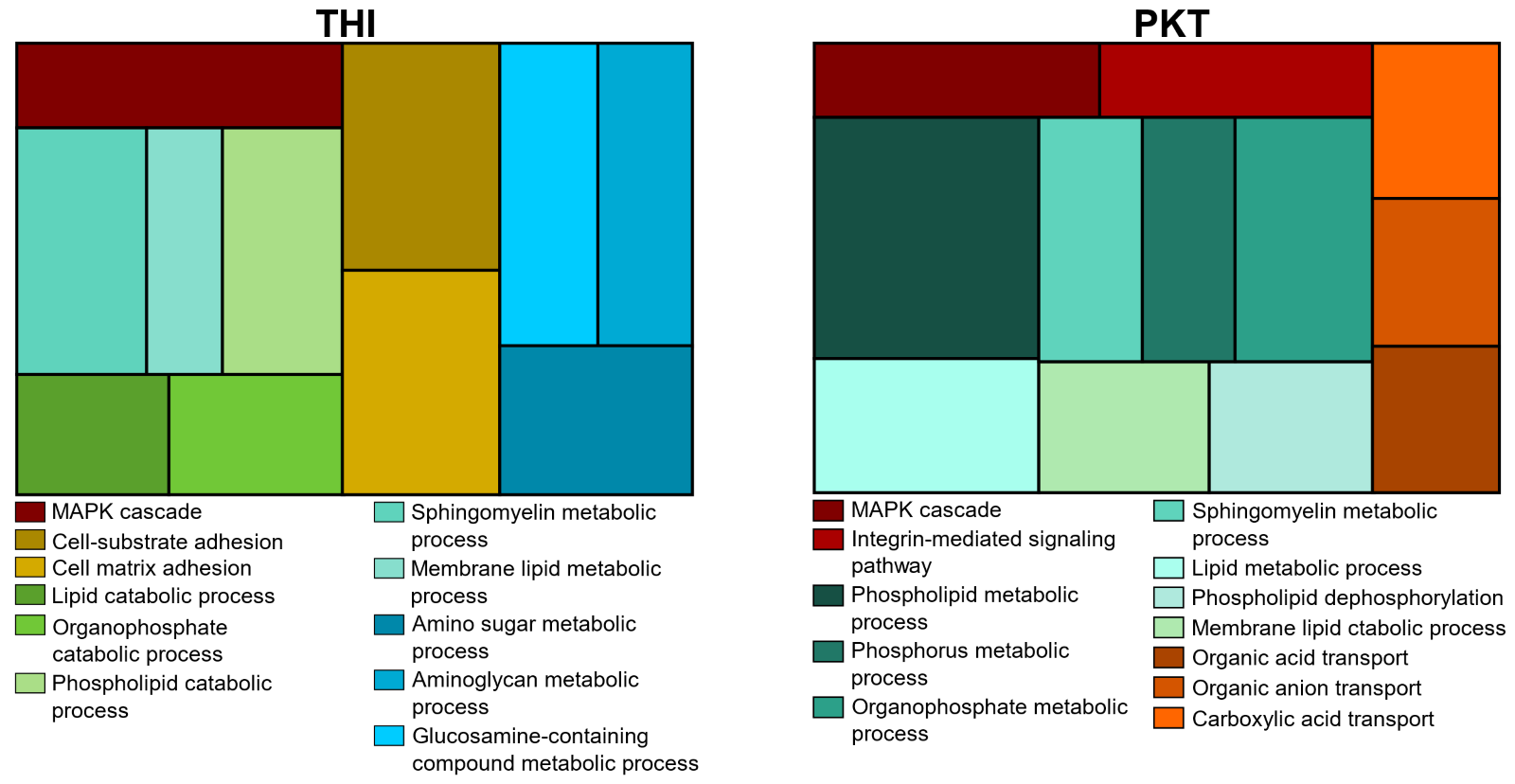


**Supplemental Figure 1**: Gene ontology (GO). TreeMap visualization of biological processes enrichment for upregulated genes with a log fold change greater than 2. Color grouping is based on higher level GO term annotation. The size of the rectangle is proportional to the number of genes in the list out of the total number of genes with similar roles in the *Ae. aegypti* genome.

**Supplemental Figure 2: Knockdown efficiency**. Mosquitoes were injected with 69 nl of dsGFP or ds*Ae-Aper50* (3 µg/µl) and knockdown was confirmed in the time points and tissues used in our assays. Total RNA was extracted from whole bodies 72 hours post injection as in the desiccation survival assay, and 96H post injection/24H post bloodmeal as in the viral challenge assay. *Ae-Aper50* transcripts and house keeping gene S7 transcripts were quantified by Rt-qPCR. Knockdown efficiency was determined by normalizing the fold change of *Ae-Aper50* relative to S7 housekeeping genes in the ds*Ae-Aper50* injected mosquitoes to dsGFP injected mosquitoes.

**Supplemental Figure 3: *Ae-Aper50* expression time course after a blood meal**. Mosquitoes were offered a bloodmeal and midguts were dissected at 4, 8, 12 and 24 hour post blood feeding, as a control unfed midgut were used. The fold change in the gene expression was calculated according to the standard DDCT method, using the unfed midgut as the control. For every mosquito line a total of 12 midguts per time points were processed.


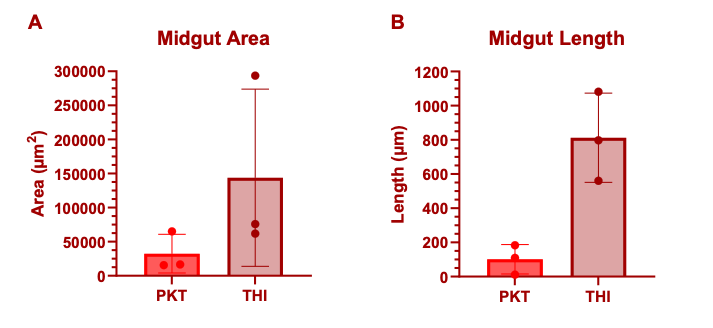


**Supplemental Figure 4: Measurements of PKT and THI midguts.** The (A) area and (B) length of the three midguts used for electron microscopy imaging of peritrophic matrix thickness were measured. Mean differences in area and length were compared by Man-Whitney Test (Area: p-value = 0.2, Length: p-value = 0.1)

**Supplemental Figure 5: ZIKV infection rates at higher bloodmeal titers.** The proportion (**A, B**) of infected *Ae. aegypti* midguts and the amount infectious virus (**C, D**) in *Ae. aegypti* midguts from the THI and PKT lines exposed to ZIKV (**A, C**) following infection with dsRNA targeting either *Ae-Aper50* or GFP as a control. Midguts were dissected 5 days post infectious bloodmeal and proportion of infected midguts and viral titers in the midgut were determined by focus forming unit assay. Virus titer used for the oral infections is displayed on the graphs.

**Supplemental Table 1: Up-regulated and Down-regulated gene.** List of genes in either the THI or PKT line that are up and down regulated following 24 hours of desiccation stress.

**Supplemental Table 2: Genes in GO analysis.** List of genes that were classified to either molecular function, cellular component, or biological process in gene ontology analysis.
