# Supplementary material for: Dehydration-induced Ae-Aper50 regulates midgut infection in Aedes aegypti mosquitoes
Source: mBio. 2025 Jan 23;16(3):e01207-24. doi: 10.1128/mbio.01207-24 (PMC11898677; doi:10.1128/mbio.01207-24)
Supplement: Supplemental Tables — Tables S1 and S2. [file mbio.01207-24-s0002.pdf]

Table 1

THI Up-Regulated					
**VB_ID**	**log2FoldChange**	**padj**	**description**		
AAEL000859	-3.605637621	1.22797E-18	unspecified product		
AAEL002467	-3.547043392	5.58561E-17	unspecified product		
AAEL006159	-3.054664706	2.18019E-06	unspecified product		
AAEL002028	-2.754919137	9.75438E-13	protein serine/threonine kinase%2C putative		
AAEL003728	-2.648075231	8.17512E-20	unspecified product		
AAEL014363	-2.500159899	1.98979E-09	unspecified product		
AAEL005293	-2.490207272	9.69337E-27	Galectin [Source:UniProtKB/TrEMBL%3BAcc:Q16ND5]		
AAEL026833	-2.446137045	7.26191E-25	unspecified product		
AAEL002217	-2.367793949	5.63676E-07	unspecified product		
AAEL012636	-2.34829885	2.58483E-15	cytochrome b5%2C putative		
AAEL022059	-2.32662829	3.28503E-07	unspecified product		
AAEL005428	-2.224415692	5.23239E-19	unspecified product		
AAEL006126	-2.196924232	6.35108E-06	unspecified product		
AAEL027610	-2.196620398	7.37382E-07	unspecified product		
AAEL008622	-2.184772881	3.28258E-17	jnk		
AAEL005432	-2.154569013	1.44351E-20	unspecified product		
AAEL006034	-2.096750711	3.30012E-07	Vanin-like protein 1 precursor%2C putative		
AAEL009111	-2.071964316	2.06396E-05	sphingomyelin phosphodiesterase		
AAEL006690	-2.065864304	1.54461E-09	ribbon		
AAEL026008	-2.056470777	8.0437E-21	unspecified product		
AAEL001988	-2.037094243	4.01487E-07	protein serine/threonine kinase%2C putative		
AAEL013350	-2.005635514	1.41334E-06	heat shock protein 26kD%2C putative		
THI Down-regulated					
**VB_ID**	**log2FoldChange**	**padj**	**description**		
AAEL001128	2.025085282	5.45599E-20	AMP dependent coa ligase		
AAEL004118	2.055648925	2.27342E-21	aldo-keto reductase		
AAEL006344	2.16034583	9.45763E-09	sulfotransferase (sult)		
AAEL002669	2.168591694	5.277E-20	AMP dependent ligase		
AAEL000678	2.205907023	6.02063E-13	alpha-amylase		
AAEL002449	2.297691659	1.57432E-09	unspecified product		
AAEL022831	2.450000484	6.05623E-06	unspecified product		
AAEL015116	2.515384034	2.71857E-12	prophenoloxidase		
AAEL013990	2.517593382	5.70707E-14	hexamerin 2 beta		
AAEL022132	2.528940642	3.1747E-09	unspecified product		
AAEL010764	2.840663807	8.09754E-12	aldehyde dehydrogenase		
AAEL022251	2.920741654	1.71261E-19	unspecified product		
PKT Up-regulated					
**VB_ID**	**log2FoldChange**	**padj**	**description**		
AAEL002467	-6.134460611	3.47229E-39	unspecified product		
AAEL002028	-3.571630947	1.54686E-18	protein serine/threonine kinase%2C putative		
AAEL014363	-3.072454637	9.91354E-15	unspecified product		
AAEL013350	-3.059925554	9.78606E-12	heat shock protein 26kD%2C putative		
AAEL000859	-2.958330225	3.94527E-13	unspecified product		
AAEL006276	-2.833421753	3.48429E-23	maltose phosphorylase		
AAEL005624	-2.815654064	9.59722E-07	unspecified product		
AAEL020330	-2.654527875	9.32684E-09	unspecified product		
AAEL003728	-2.64661908	2.77612E-19	unspecified product		
AAEL005428	-2.459868088	2.36118E-22	unspecified product		
AAEL022253	-2.456360073	1.26659E-07	unspecified product		
AAEL000323	-2.425169003	6.85657E-05	cysteine-rich venom protein%2C putative		
AAEL027610	-2.411463028	3.15763E-07	unspecified product		
AAEL007344	-2.390365607	6.20134E-17	LITAF domain-containing protein [Source:UniProtKB/TrEMBL%3BAcc:A0A1S4FG64]		
AAEL019751	-2.369821231	5.93882E-14	unspecified product		
AAEL001988	-2.306799329	4.9894E-07	protein serine/threonine kinase%2C putative		
AAEL008622	-2.298273565	1.85798E-19	jnk		
AAEL002908	-2.284181728	1.24994E-05	unspecified product		
AAEL014541	-2.284179714	7.06448E-24	maltose phosphorylase		
AAEL002004	-2.279975221	3.43662E-05	protein serine/threonine kinase%2C putative		

AAEL003632	-2.223028972	4.38386E-14	Clip-Domain Serine Protease family B.		
AAEL028247	-2.216055754	2.8279E-09	unspecified product		
AAEL002138	-2.200922538	3.23327E-06	triacylglycerol lipase%2C putative		
AAEL017975	-2.195010013	1.88424E-09	heat shock protein HSP70		
AAEL025839	-2.172706632	3.43276E-11	unspecified product		
AAEL006479	-2.139620092	1.10255E-16	unspecified product		
AAEL015451	-2.129698752	0.000142908	unspecified product		
AAEL027970	-2.119844964	1.57947E-17	unspecified product		
AAEL017380	-2.115023248	3.06127E-11	unspecified product		
AAEL017136	-2.110398005	4.40457E-20	cytochrome P450		
AAEL006034	-2.091893787	2.09637E-06	Vanin-like protein 1 precursor%2C putative		
AAEL021795	-2.08159442	2.35076E-15	unspecified product		
AAEL011038	-2.080630068	1.28185E-26	integrin alpha-ps		
AAEL001367	-2.079079051	3.27792E-13	type IV inositol 5-phosphatase		
AAEL010068	-2.077815884	1.82526E-11	unspecified product		
AAEL004277	-2.077393766	4.24421E-15	unspecified product		
AAEL013348	-2.066232467	3.90784E-07	lethal(2)essential for life protein%2C l2efl		
AAEL007765	-2.059036337	4.53193E-24	Serine Protease Inhibitor (serpin) likely cleavage at K/R. Transcript A.		
AAEL026008	-2.058711781	1.88857E-20	unspecified product		
AAEL006883	-2.039758152	5.88797E-10	unspecified product		
AAEL013345	-2.031469477	1.09903E-07	alphaA-crystallin%2C putative		
AAEL003881	-2.030545162	2.69744E-06	ubiquitin%2C putative		
AAEL002413	-2.023104513	8.158E-12	sphingomyelin phosphodiesterase		
AAEL007094	-2.019685501	6.95199E-10	4-nitrophenylphosphatase [Source:UniProtKB/TrEMBL%3BAcc:Q0IF20]		
AAEL025022	-2.007083314	3.94573E-05	unspecified product		
AAEL003738	-2.002731511	3.36227E-11	unspecified product		
AAEL011598	-2.001386379	2.92515E-09	Gustatory receptor [Source:UniProtKB/TrEMBL%3BAcc:A0A1S4FU22]		
PKT Down-regulated					
**VB_ID**	**log2FoldChange**	**padj**	**description**		
AAEL012693	2.528580218	2.34376E-09	tripartite motif protein		
Shared Up-regulated					
Gene					
AAEL002467					
AAEL002028					
AAEL014363					
AAEL013350					
AAEL000859					
AAEL003728					
AAEL005428					
AAEL027610					
AAEL001988					
AAEL008622					
AAEL006034					
AAEL026008					

**Table 2**										
THI Up-Regulated										
ID	Name	Bgd count	Result count	Result gene list	Pct of bgd	Fold enrichm	Odds ratio	P-value	Benjamini	Bonferroni
GO:0031589	cell-substrate adhesion	2	1	AAEL005432	50	426.81	923.58	0.00234171	0.04427504	0.15221106
GO:0007160	cell-matrix adhesion	2	1	AAEL005432	50	426.81	923.58	0.00234171	0.04427504	0.15221106
GO:0006685	sphingomyelin catabolic process	4	1	AAEL009111	25	213.4	307.81	0.00467835	0.04427504	0.30409305
GO:0006684	sphingomyelin metabolic process	4	1	AAEL009111	25	213.4	307.81	0.00467835	0.04427504	0.30409305
GO:0006030	chitin metabolic process	104	2	AAEL002467,AA	1.9	16.42	19.58	0.00634349	0.04427504	0.41232662
GO:1901071	glucosamine-containing compound metabol	106	2	AAEL002467,AA	1.9	16.11	19.2	0.00658232	0.04427504	0.4278511
GO:0006040	amino sugar metabolic process	108	2	AAEL002467,AA	1.9	15.81	18.83	0.00682525	0.04427504	0.44364105
GO:0009395	phospholipid catabolic process	6	1	AAEL009111	16.7	142.27	184.65	0.00700995	0.04427504	0.45564661
GO:0006022	aminoglycan metabolic process	116	2	AAEL002467,AA	1.7	14.72	17.5	0.00783743	0.04427504	0.50943297
GO:0000165	MAPK cascade	7	1	AAEL008622	14.3	121.95	153.86	0.00817385	0.04427504	0.53130044
GO:0030149	sphingolipid catabolic process	7	1	AAEL009111	14.3	121.95	153.86	0.00817385	0.04427504	0.53130044
GO:0046466	membrane lipid catabolic process	7	1	AAEL009111	14.3	121.95	153.86	0.00817385	0.04427504	0.53130044
GO:0006665	sphingolipid metabolic process	13	1	AAEL009111	7.7	65.66	76.89	0.01513088	0.0756544	0.98350715
GO:0044242	cellular lipid catabolic process	15	1	AAEL009111	6.7	56.91	65.89	0.01743986	0.08097077	1

GO:0046434	organophosphate catabolic process	25	1	AAEL009111	4	34.14	38.4	0.02890999	0.12527663	1
GO:0006643	membrane lipid metabolic process	37	1	AAEL009111	2.7	23.07	25.57	0.04251096	0.17270077	1
GO:0016042	lipid catabolic process	40	1	AAEL009111	2.5	21.34	23.6	0.04588361	0.17543735	1
PKT Up-Regulated										
ID	Name	Bgd count	Result count	Result gene list	Pct of bgd	Fold enrichm	Odds ratio	P-value	Benjamini	Bonferroni
GO:0006644	phospholipid metabolic process	87	4	AAEL001367,AA	4.6	18.9	23.02	5.39E-05	0.00630579	0.00630579
GO:0044255	cellular lipid metabolic process	169	4	AAEL001367,AA	2.4	9.73	11.49	0.00069297	0.04053885	0.0810777
GO:0019637	organophosphate metabolic process	231	4	AAEL001367,AA	1.7	7.12	8.31	0.00220338	0.08593191	0.25779573
GO:0006643	membrane lipid metabolic process	37	2	AAEL002413,AA	5.4	22.22	25.22	0.00360276	0.09666807	0.42152345
GO:0006629	lipid metabolic process	275	4	AAEL001367,AA	1.5	5.98	6.93	0.00413111	0.09666807	0.48334033
GO:0046488	phosphatidylinositol metabolic process	47	2	AAEL001367,AA	4.3	17.49	19.6	0.0057609	0.09820779	0.67402509
GO:0006650	glycerophospholipid metabolic process	56	2	AAEL001367,AA	3.6	14.68	16.32	0.00809739	0.09820779	0.94739405
GO:0046486	glycerolipid metabolic process	57	2	AAEL001367,AA	3.5	14.42	16.02	0.00837931	0.09820779	0.98037966
GO:0006685	sphingomyelin catabolic process	4	1	AAEL002413	25	102.75	141.88	0.0096982	0.09820779	1
GO:0006684	sphingomyelin metabolic process	4	1	AAEL002413	25	102.75	141.88	0.0096982	0.09820779	1
GO:0009395	phospholipid catabolic process	6	1	AAEL002413	16.7	68.5	85.12	0.01451328	0.09820779	1
GO:0006796	phosphate-containing compound metabolic	618	5	AAEL001367,AA	0.8	3.33	3.88	0.01533145	0.09820779	1
GO:0006793	phosphorus metabolic process	622	5	AAEL001367,AA	0.8	3.3	3.85	0.01572991	0.09820779	1
GO:0046466	membrane lipid catabolic process	7	1	AAEL002413	14.3	58.71	70.92	0.01691235	0.09820779	1
GO:0030149	sphingolipid catabolic process	7	1	AAEL002413	14.3	58.71	70.92	0.01691235	0.09820779	1
GO:0000165	MAPK cascade	7	1	AAEL008622	14.3	58.71	70.92	0.01691235	0.09820779	1
GO:0046856	phosphatidylinositol dephosphorylation	7	1	AAEL001367	14.3	58.71	70.92	0.01691235	0.09820779	1
GO:0032309	icosanoid secretion	8	1	AAEL019751	12.5	51.38	60.79	0.0193058	0.09820779	1
GO:0007229	integrin-mediated signaling pathway	8	1	AAEL011038	12.5	51.38	60.79	0.0193058	0.09820779	1
GO:0071715	icosanoid transport	8	1	AAEL019751	12.5	51.38	60.79	0.0193058	0.09820779	1
GO:0046839	phospholipid dephosphorylation	8	1	AAEL001367	12.5	51.38	60.79	0.0193058	0.09820779	1
GO:0050482	arachidonic acid secretion	8	1	AAEL019751	12.5	51.38	60.79	0.0193058	0.09820779	1
GO:1903963	arachidonate transport	8	1	AAEL019751	12.5	51.38	60.79	0.0193058	0.09820779	1
GO:0015909	long-chain fatty acid transport	9	1	AAEL019751	11.1	45.67	53.18	0.02169364	0.10152625	1
GO:0015908	fatty acid transport	9	1	AAEL019751	11.1	45.67	53.18	0.02169364	0.10152625	1
GO:0046942	carboxylic acid transport	10	1	AAEL019751	10	41.1	47.27	0.02407588	0.10432882	1
GO:0015718	monocarboxylic acid transport	10	1	AAEL019751	10	41.1	47.27	0.02407588	0.10432882	1
GO:0015711	organic anion transport	12	1	AAEL019751	8.3	34.25	38.67	0.02882361	0.12044153	1
GO:0006665	sphingolipid metabolic process	13	1	AAEL002413	7.7	31.62	35.44	0.03118913	0.12163762	1
GO:0015849	organic acid transport	13	1	AAEL019751	7.7	31.62	35.44	0.03118913	0.12163762	1
GO:0030258	lipid modification	14	1	AAEL001367	7.1	29.36	32.71	0.0335491	0.1266208	1
GO:0044242	cellular lipid catabolic process	15	1	AAEL002413	6.7	27.4	30.37	0.03590353	0.13127229	1
THI Down-Regulated										
ID	Name	Bgd count	Result count	Result gene list	Pct of bgd	Fold enrichm	Odds ratio	P-value	Benjamini	Bonferroni
GO:0009397	folic acid-containing compound catabolic pro	1	1	AAEL010764,	100	1109.7	inf	0.00090114	0.0096873	0.03874921
GO:0009258	10-formyltetrahydrofolate catabolic process	1	1	AAEL010764,	100	1109.7	inf	0.00090114	0.0096873	0.03874921
GO:0009256	10-formyltetrahydrofolate metabolic proces	1	1	AAEL010764,	100	1109.7	inf	0.00090114	0.0096873	0.03874921
GO:0042560	pteridine-containing compound catabolic pro	1	1	AAEL010764,	100	1109.7	inf	0.00090114	0.0096873	0.03874921
GO:0043649	dicarboxylic acid catabolic process	2	1	AAEL010764,	50	554.85	1231.78	0.00180156	0.0154934	0.07746699
GO:0046653	tetrahydrofolate metabolic process	6	1	AAEL010764,	16.7	184.95	246.27	0.00539591	0.03006503	0.23202428
GO:0006730	one-carbon metabolic process	7	1	AAEL010764,	14.3	158.53	205.2	0.00629268	0.03006503	0.27058525
GO:0043648	dicarboxylic acid metabolic process	7	1	AAEL010764,	14.3	158.53	205.2	0.00629268	0.03006503	0.27058525
GO:0006760	folic acid-containing compound metabolic p	7	1	AAEL010764,	14.3	158.53	205.2	0.00629268	0.03006503	0.27058525
GO:0042219	cellular modified amino acid catabolic proce	9	1	AAEL010764,	11.1	123.3	153.88	0.00808403	0.03476133	0.34761334
GO:0042558	pteridine-containing compound metabolic p	10	1	AAEL010764,	10	110.97	136.77	0.00897862	0.03509823	0.3860805
GO:0055114	obsolete oxidation-reduction process	671	3	AAEL004118,AA	0.4	4.96	6.68	0.0191767	0.06871653	0.8245983
GO:0006575	cellular modified amino acid metabolic proc	24	1	AAEL010764,	4.2	46.24	53.45	0.0214268	0.06901753	0.92135222
GO:0016054	organic acid catabolic process	27	1	AAEL010764,	3.7	41.1	47.27	0.02407588	0.06901753	1
GO:0046395	carboxylic acid catabolic process	27	1	AAEL010764,	3.7	41.1	47.27	0.02407588	0.06901753	1
GO:0044282	small molecule catabolic process	45	1	AAEL010764,	2.2	24.66	27.89	0.03983532	0.10705742	1
PKT Down-regulated										
N/A										
